# Automated classification of sagittal, vertical, and transverse malocclusions from 3D intraoral scans via a multi-head CNN framework

**DOI:** 10.1007/s10266-026-01400-9

**Published:** 2026-05-13

**Authors:** Yunus Balel, Kaan Sağtaş, Fatih Teke, Mehmet Ali Kurt

**Affiliations:** 1 Department of Oral and Maxillofacial Surgery, Faculty of Dentistry, Sivas Cumhuriyet University, Merkez, 58140 Sivas, Turkey; 2 SEMRUK Technology Inc., Cumhuriyet Teknokent, Sivas, Turkey; 3 Department of Industrial Engineering, Sivas Cumhuriyet University, Sivas, Turkey; 4 Department of Computer Engineering, Yildiz Technical University, Istanbul, Turkey; 5 Department of Machine Engineering, Sivas Cumhuriyet University, Sivas, Turkey

**Keywords:** Deep learning, Malocclusion classification, Intraoral scanning, Multi-task learning

## Abstract

This study aimed to develop and evaluate a multi-task deep learning model for the simultaneous classification of sagittal, vertical, and transverse malocclusion components, as well as midline deviation, using three-dimensional (3D) intraoral scan (IOS) data. A total of 801 anonymized 3D IOS datasets were retrospectively collected. Surface mesh (STL) files were standardized and converted into six-view two-dimensional (2D) representations. This approach preserved morphological information while enabling the use of transfer learning-based convolutional neural networks (CNNs). A shared-backbone, multi-head architecture was designed to perform simultaneous classification tasks. Six CNN backbones were evaluated using accuracy, precision, recall, F1-score, and ROC-AUC. EfficientNetV2B3 achieved the highest performance in anterior bite classification (accuracy: 80%, ROC-AUC ≈ 0.89). ResNet50 provided the most balanced results for sagittal and midline classification. Transverse occlusion classification showed comparatively lower performance across all models. Multi-view representations derived from 3D intraoral scans enable simultaneous and clinically relevant classification of multiple malocclusion components. While strong performance was observed for anterior bite and midline tasks, sagittal and transverse classifications remain more challenging. IOS-based multi-task AI systems may serve as supportive tools in orthodontic diagnosis and digital workflows.

**Supplementary Information:**

The online version contains supplementary material available at https://doi.org/10.1007/s10266-026-01400-9.

## Introduction

Malocclusion is a significant oral health problem with a high global prevalence, characterized by disturbances in the sagittal, vertical, and transverse relationships between the teeth and jaws [1, 2]. In addition to aesthetic concerns, it is associated with various health complications, including impaired masticatory function, speech difficulties, temporomandibular joint (TMJ) disorders, and an increased risk of periodontal disease [3, 4]. Traditionally, the diagnosis of malocclusion has relied on manual evaluation by orthodontists using clinical examination, cephalometric analysis, dental plaster models, and photographic records [5]. However, these manual processes are inherently time-consuming, prone to human error, and susceptible to inter-clinician variability.

In recent years, artificial intelligence (AI) has ushered in a new era of precision and efficiency in healthcare, particularly in complex pattern recognition and decision-making processes [6, 7]. In orthodontics, AI applications have achieved notable advances in areas such as the identification of cephalometric landmarks, acceleration of treatment planning, and prediction of treatment outcomes. Deep learning techniques enable more consistent and accurate decision-making by reducing human subjectivity [8]. Numerous studies have reported the classification of malocclusion types using intraoral photographs and radiographs, with these models frequently achieving accuracy rates exceeding 90% [2, 9–11]. 

Despite these advances, the majority of existing studies have focused predominantly on two-dimensional (2D) images, such as photographs and radiographs [12]. However, 2D images may be insufficient to capture the complex three-dimensional morphology and critical occlusal relationships between the jaws that define malocclusion. Although the use of three-dimensional (3D) intraoral scanners (IOS) has become increasingly widespread with the development of digital dentistry, the automatic determination of occlusal classifications based on these data remains insufficiently explored. In particular, the direct prediction of sagittal, vertical, and transverse anomalies from 3D point clouds or mesh data represents a critical gap in the digital transformation of orthodontic diagnosis.

The aim of this study is to develop and evaluate an AI-based model capable of automatically classifying malocclusion types from three-dimensional intraoral scan images. The proposed model can simultaneously identify molar relationships according to the Angle classification (Class I, II, III), vertical occlusal conditions (normal bite, deep bite, open bite), transverse occlusal relationships (normal transverse occlusion, crossbite), and midline alignment (normal or deviated). This approach is expected to accelerate clinical workflows, provide an objective diagnostic tool, and enhance precision in orthodontic treatment planning.

## Materials and methods

The study protocol was reviewed and approved by the Sivas Cumhuriyet University Health Sciences Research Ethics Committee with the approval number 25_08_18_4. All data used in the study were anonymized, and no information that could reveal patient identity was included in the analysis process.

### Dataset and sources

The dataset used in this study was created by combining three-dimensional intraoral scan data obtained from two different sources. The first data group consisted of digital models acquired using intraoral scanners from individuals who presented to the Sivas Cumhuriyet University, with complaints of dentofacial deformity and temporomandibular joint pain. The second data group was obtained from the Bits2Bites open-access dataset published by Borghi et al. [13] This approach aimed to ensure that the dataset both reflected clinical reality and was comparable with existing literature.

All scans were obtained in occlusion and consisted of surface meshes (STL format) covering both the maxilla and mandible. The dataset was constructed to include samples acquired using different scanner systems, with the aim of reflecting device-related variations in the model and thereby enhancing its generalization capability.

### Inclusion and exclusion criteria

The dataset included intraoral scans obtained from patients with permanent dentition and sufficient image quality to allow reliable occlusal assessment. Scans with mixed dentition were excluded to ensure consistency in occlusal classification. Patients with a history of previous orthodontic treatment were excluded to avoid treatment-induced alterations in occlusal relationships. Additionally, scans presenting severe artifacts, incomplete dental arches, or significant distortions that could compromise anatomical interpretation were excluded from the study. Cases with known craniofacial syndromes or severe developmental anomalies were also excluded, as these conditions may introduce atypical morphological patterns beyond standard malocclusion classifications.

### Data preprocessing and labeling

Before model training, the three-dimensional scan data underwent a standardized preprocessing pipeline. All STL models were aligned and normalized to a common reference coordinate system to preserve occlusal relationships. Maxillary and mandibular models were merged in the occlusal position and processed while maintaining anatomical integrity.

Each patient’s data were labeled using a clinically meaningful multi-label classification scheme. Sagittal molar relationships were assessed separately for the right and left sides according to Angle’s standard classification as Class I, Class II, or Class III. Vertical anterior relationships were classified as normal bite, deep bite, or open bite. Transverse occlusal relationships were defined as normal or crossbite, and midline status was labeled as centered or deviated.

The classification of malocclusion types was performed based on standard orthodontic diagnostic principles. Sagittal molar relationships were determined according to Angle’s classification by evaluating the relationship between the mesiobuccal cusp of the maxillary first molar and the buccal groove of the mandibular first molar [14]. Vertical relationships were assessed based on the degree of vertical overlap between the maxillary and mandibular anterior teeth. Deep bite was defined as excessive vertical overlap exceeding one-third of the clinical crown height of the mandibular incisors [15]. Open bite was defined as the absence of vertical overlap between the anterior teeth. Cases within normal limits were classified as normal bite. Transverse relationships were defined based on the presence or absence of crossbite, determined by buccolingual discrepancies between the maxillary and mandibular teeth. Midline deviation was assessed by evaluating the alignment between the maxillary and mandibular dental midlines.

After labeling, the data were split into training (60%), validation (20%), and test (20%) sets. To prevent data leakage, dataset splitting was strictly performed at the patient level. All multi-view representations generated from a single STL file were kept within the same subset (training, validation, or test). This ensured that no views originating from the same patient appeared across different data splits, thereby avoiding information leakage and ensuring a fair evaluation of model performance.

### Conversion of three-dimensional data to multi-view representations

**Fig. 1:**
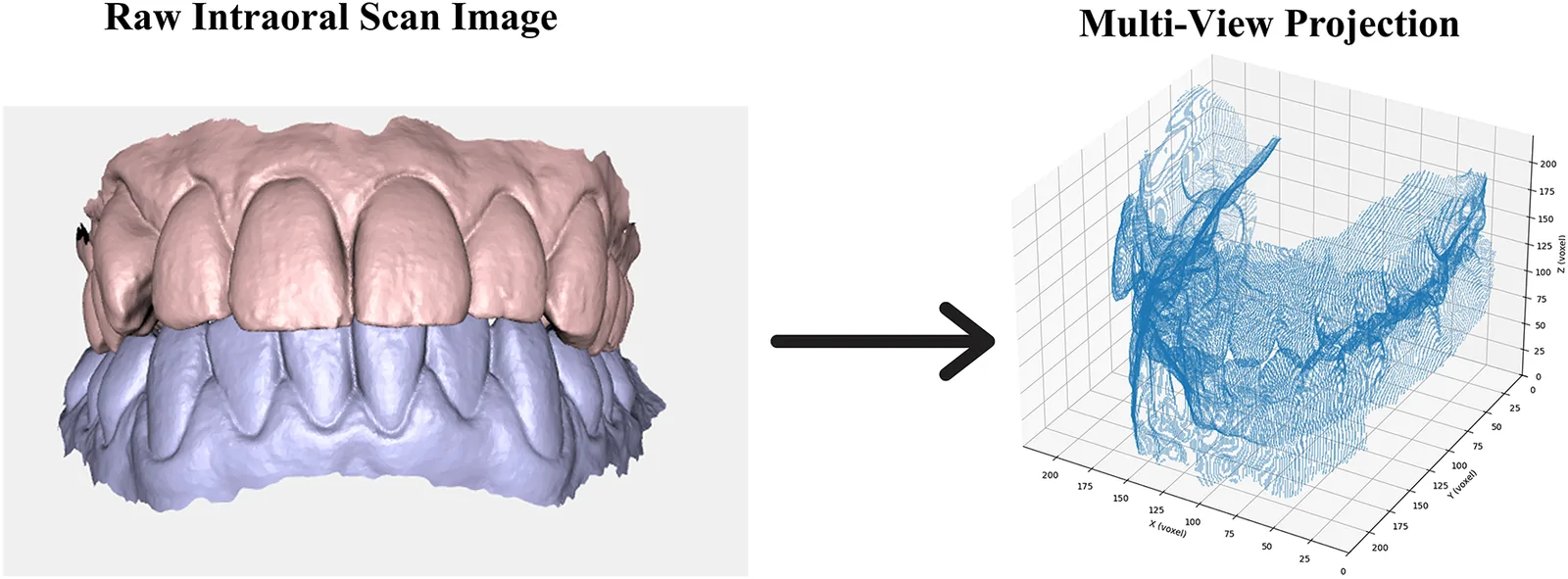
Overview of the multi-view representation pipeline used to convert three-dimensional intraoral scan (STL) data into multiple two-dimensional anatomical views for deep learning-based classification

Direct processing of raw three-dimensional STL data with deep learning models presents significant disadvantages, including high computational cost and inefficient memory usage. Therefore, in this study, three-dimensional intraoral scan data were converted into a two-dimensional “multi-view” representation (Fig. 1). As illustrated in Fig. 1, raw intraoral scan data were first processed into standardized 3D representations and subsequently transformed into structured multi-view projections. This pipeline enables the preservation of anatomical relationships while converting complex 3D data into a format suitable for CNN-based analysis. This approach aims to preserve three-dimensional geometric information while avoiding the computational burden associated with voxel-based representations.

Instead of a single three-dimensional input of size 224 × 224 × 224, an input structure of size 6 × 224 × 224 × 3 was used, consisting of six different views, each with a resolution of 224 × 224 and three color channels. This strategy reduced the model input size from approximately 11.2 million pixels to 903 thousand pixels, significantly improving computational efficiency. Moreover, this representation enabled the use of transfer learning-based CNN architectures with proven effectiveness in image processing tasks. The selection of six views was based on the need to capture clinically relevant anatomical perspectives, including frontal, posterior, sagittal (right and left), and occlusal views. These views were considered sufficient to represent key occlusal relationships while maintaining computational efficiency. Increasing the number of views would lead to higher computational cost with potentially limited additional diagnostic information, whereas fewer views might fail to capture critical morphological features.

**Fig. 2:**
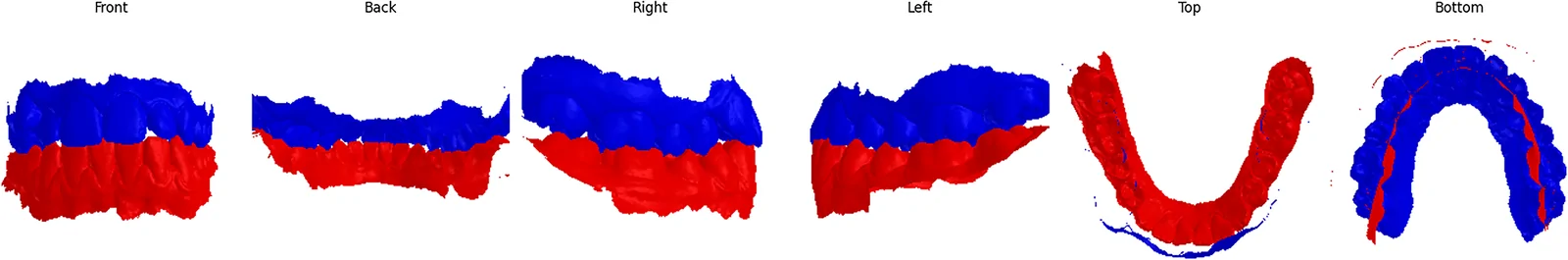
Examples of standardized multi-view images generated from intraoral scan data, including frontal, posterior, right sagittal, left sagittal, and occlusal views. The maxilla and mandible are color-coded to enhance morphological discrimination

To enhance morphological discrimination, the maxilla and mandible were color-coded differently, and standard anatomical views representing frontal, posterior, right sagittal, left sagittal, and occlusal perspectives were obtained in a virtual imaging environment. All images were normalized to a fixed resolution and prepared for model input (Fig. 2).

### Model architecture

In this study, a multi-task convolutional neural network architecture capable of simultaneously processing multi-view images was developed. The model processes images obtained from six different anatomical views of each patient in parallel and produces concurrent outputs for multiple clinical classification tasks.

**Fig. 3:**
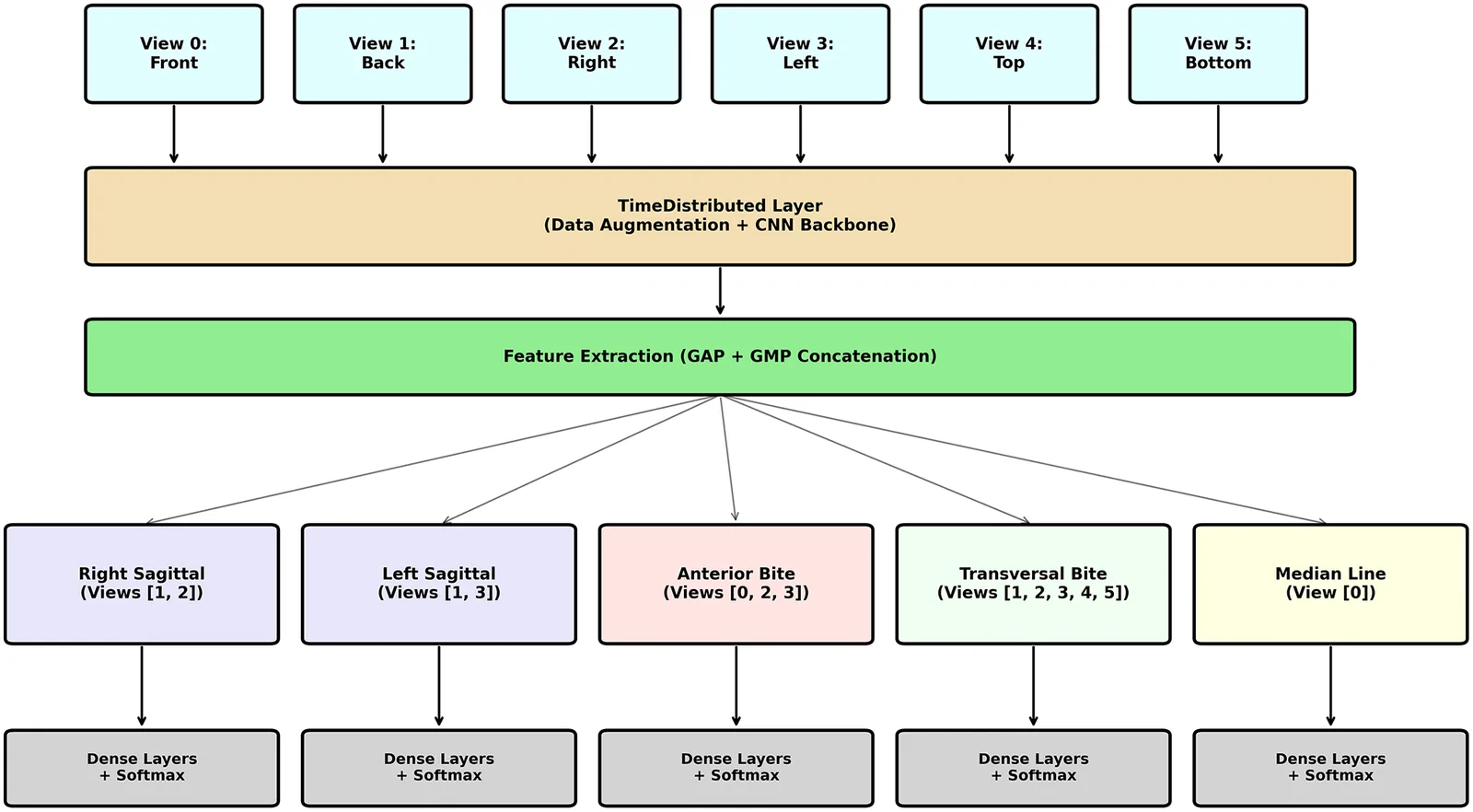
Schematic illustration of the proposed multi-task convolutional neural network architecture. Multi-view images are processed through a shared CNN backbone, followed by task-specific classification heads for sagittal, vertical, transverse, and midline assessments

At the input stage, all views were subjected to feature extraction through a single CNN backbone with shared weights. This approach enables the model to learn a consistent representation of morphological information derived from different viewpoints. The extracted features were then directed to multiple task-specific decision heads (multi-heads) defined for each clinical task (Fig. 3).

### Model training and hyperparameters

During model training, the dataset was divided into training, validation, and test subsets, with careful attention paid to maintaining balanced class distributions across all subsets. To improve the generalization ability of the model, extensive data augmentation techniques were applied during training. These included random rotations (± 15°), scaling (0.9–1.1), translations (up to 10% of image dimensions), brightness and contrast adjustments (± 20%), and the addition of Gaussian noise.

The batch size was set to 16 during training. The AdamW optimizer was used with an initial learning rate of 0.0003. To mitigate the effects of class imbalance, Categorical Focal Crossentropy was selected as the loss function. The training process was planned for a maximum of 150 epochs; however, early stopping was applied if no improvement in validation loss was observed for 20 consecutive epochs.

### CNN backbone models

Models were constructed using six different CNN backbone architectures with distinct architectural characteristics: VGG16, ResNet50, MobileNetV2, EfficientNetB3, EfficientNetV2B3, and ConvNeXtTiny. All backbone models were integrated into the framework using a transfer learning approach with weights pre-trained on the ImageNet dataset. Apart from the backbone architectures, all model structures, training protocols, and hyperparameters were kept constant to ensure that performance differences could be attributed solely to the backbone architecture.

### Statistical analysis

The performance of the developed deep learning model was evaluated on the test dataset, which was not used during training. Classification performance was analyzed using accuracy, recall (sensitivity), precision, specificity, and F1-score metrics. To reduce the impact of the multi-class and class-imbalanced structure, all metrics were calculated using a macro-average approach. A macro-average approach was preferred to ensure that all classes contributed equally to the evaluation, regardless of class imbalance, and to avoid dominance of majority classes in the reported performance metrics.

The discriminative ability of the model was assessed using receiver operating characteristic (ROC) curves and the area under the curve (ROC-AUC). For multi-class classification tasks, the one-versus-rest (OvR) approach was adopted for ROC-AUC calculation. Performance comparisons among different CNN backbone architectures were conducted by evaluating all models using the same data split and training protocol.

To examine the classification behavior of the model in detail, confusion matrices were generated for each classification task. All statistical analyses were performed using the Python programming language.

## Results

Data from a total of 801 patients were used in this study and were divided into training (60%, *n* = 480), validation (20%, *n* = 161), and test (20%, *n* = 160) sets. The distribution of classes within the dataset is presented in Table 1.

**Table 1:** Distribution of malocclusion classes across the dataset, including sagittal molar relationships (right and left), anterior bite types, transverse occlusal relationships, and midline status in the training, validation, and test subsets

Projection	Case	Total	Train	Validation	Test
Right Sagittal	Class I	304	183	60	61
	Class II	290	174	58	58
	Class III	207	123	43	41
Left Sagittal	Class I	320	192	64	64
	Class II	275	165	55	55
	Class III	206	123	42	41
Anterior Bite	Normal	267	159	55	53
	Deep Bite	290	174	58	58
	Open Bite	244	147	48	49
Transversal Bite	Normal	180	108	36	36
	Cross Bite	621	372	125	124
Midline	Deviated	184	111	36	37
	Normal	617	369	125	123
Total		801	480	161	160

### Overall model performance

The performance metrics of the models constructed using different CNN backbone architectures on the test dataset are presented in detail in Table 2. Overall, model performance was found to vary depending on both the specific classification task and the backbone architecture employed.

**Table 2:** Performance metrics of the multi-task classification models using different CNN backbone architectures on the test dataset

Projection	Accuracy	ROC-AUC	Recall	Precision	Specificity	F1-Score
*ResNet50*						
Right Sagittal	0.71	0.80	0.74	0.71	0.85	0.71
Left Sagittal	0.69	0.78	0.72	0.69	0.84	0.69
Anterior Bite	0.65	0.73	0.65	0.71	0.82	0.65
Midline	0.78	0.72	0.83	0.88	0.88	0.85
Transversal Bite	0.63	0.50	0.73	0.77	0.77	0.75
*VGG16*						
Right Sagittal	0.72	0.86	0.65	0.80	0.82	0.70
Left Sagittal	0.36	0.53	0.33	0.33	0.66	0.32
Anterior Bite	0.56	0.86	0.58	0.67	0.79	0.54
Midline	0.76	0.56	0.55	0.64	0.55	0.55
Transversal Bite	0.68	0.68	0.64	0.62	0.64	0.63
*MobileNetv2*						
Right Sagittal	0.68	0.73	0.62	0.63	0.80	0.62
Left Sagittal	0.48	0.55	0.37	0.37	0.72	0.37
Anterior Bite	0.44	0.76	0.49	0.57	0.72	0.45
Midline	0.80	0.65	0.58	0.89	0.58	0.58
Transversal Bite	0.56	0.64	0.60	0.58	0.60	0.54
*EfficientNetB3*						
Right Sagittal	0.52	0.78	0.65	0.55	0.71	0.58
Left Sagittal	0.56	0.70	0.59	0.54	0.77	0.53
Anterior Bite	0.60	0.79	0.51	0.76	0.76	0.50
Midline	0.76	0.72	0.67	0.67	0.67	0.67
Transversal Bite	0.44	0.45	0.43	0.44	0.43	0.41
*EfficientNetv2B3*						
Right Sagittal	0.68	0.86	0.63	0.60	0.82	0.60
Left Sagittal	0.48	0.61	0.48	0.48	0.73	0.46
Anterior Bite	0.80	0.89	0.78	0.89	0.88	0.80
Midline	0.72	0.60	0.53	0.55	0.53	0.52
Transversal Bite	0.48	0.51	0.42	0.43	0.42	0.42
*ConvNeXtTiny*						
Right Sagittal	0.16	0.51	0.25	0.14	0.63	0.13
Left Sagittal	0.44	0.69	0.33	0.47	0.67	0.25
Anterior Bite	0.44	0.72	0.37	0.47	0.67	0.34
Midline	0.40	0.57	0.60	0.64	0.60	0.39
Transversal Bite	0.52	0.48	0.49	0.49	0.49	0.47

Metrics include accuracy, precision, recall (sensitivity), specificity, F1-score, and ROC-AUC for each classification task

**Fig. 4:**
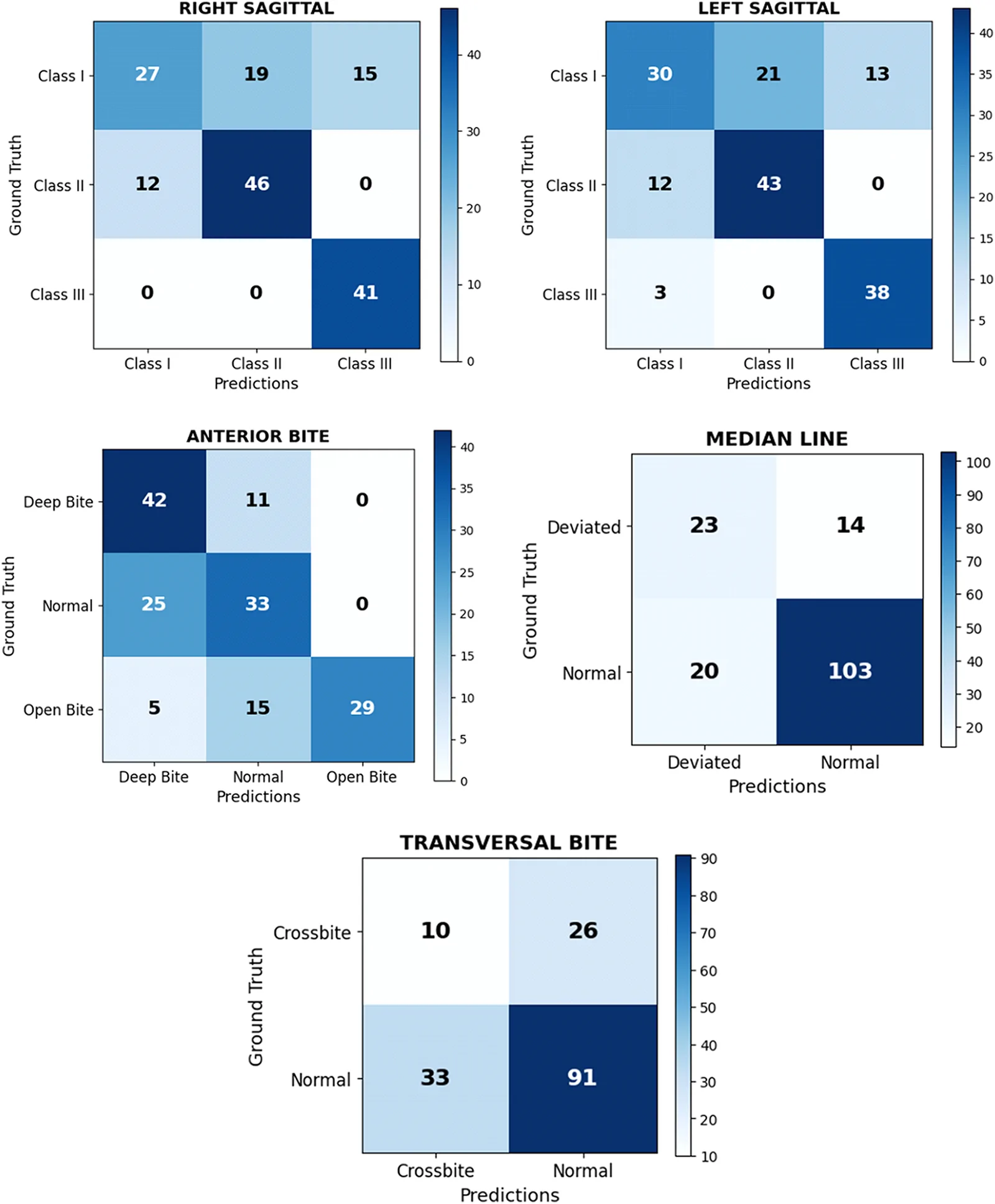
Confusion matrices of the best-performing model (ResNet50) for sagittal molar relationship classification across different projections, demonstrating class-wise prediction performance on the test dataset

In sagittal molar relationship classification (right and left), the ResNet50 and VGG16 architectures demonstrated relatively higher accuracy and F1-score values, whereas the ConvNeXtTiny architecture exhibited the lowest performance in both sagittal tasks. The confusion matrices of the overall best-performing model, ResNet50, across different projections are shown in Fig. 4. As shown in Fig. 4, the confusion matrices indicate that the model performs well in correctly identifying Class III cases, while misclassifications are more frequently observed between Class I and Class II categories. This pattern reflects the greater morphological similarity between these classes and highlights the relative difficulty of borderline sagittal classification.

Figure S1 illustrates the changes in training and validation losses across epochs for each output of the multi-head model (right/left sagittal, anterior bite, transversal bite, and midline). Across all heads, training loss decreased rapidly during the initial epochs and converged to a low and stable level after approximately 20–30 epochs, indicating effective learning from the training data. In contrast, validation losses exhibited notable fluctuations, particularly during the early epochs, but showed a more stable plateau behavior after approximately 30–40 epochs. The persistent gap between training and validation losses suggests mild to moderate overfitting. When comparing tasks, the validation losses for the right sagittal and anterior bite outputs were relatively higher, indicating that these tasks were more challenging, whereas the midline and transversal bite outputs demonstrated lower and more stable validation losses, reflecting better generalization. Overall, the absence of meaningful improvement in validation losses beyond 40–50 epochs suggests that terminating training via early stopping within this range would be appropriate.

**Fig. 5:**
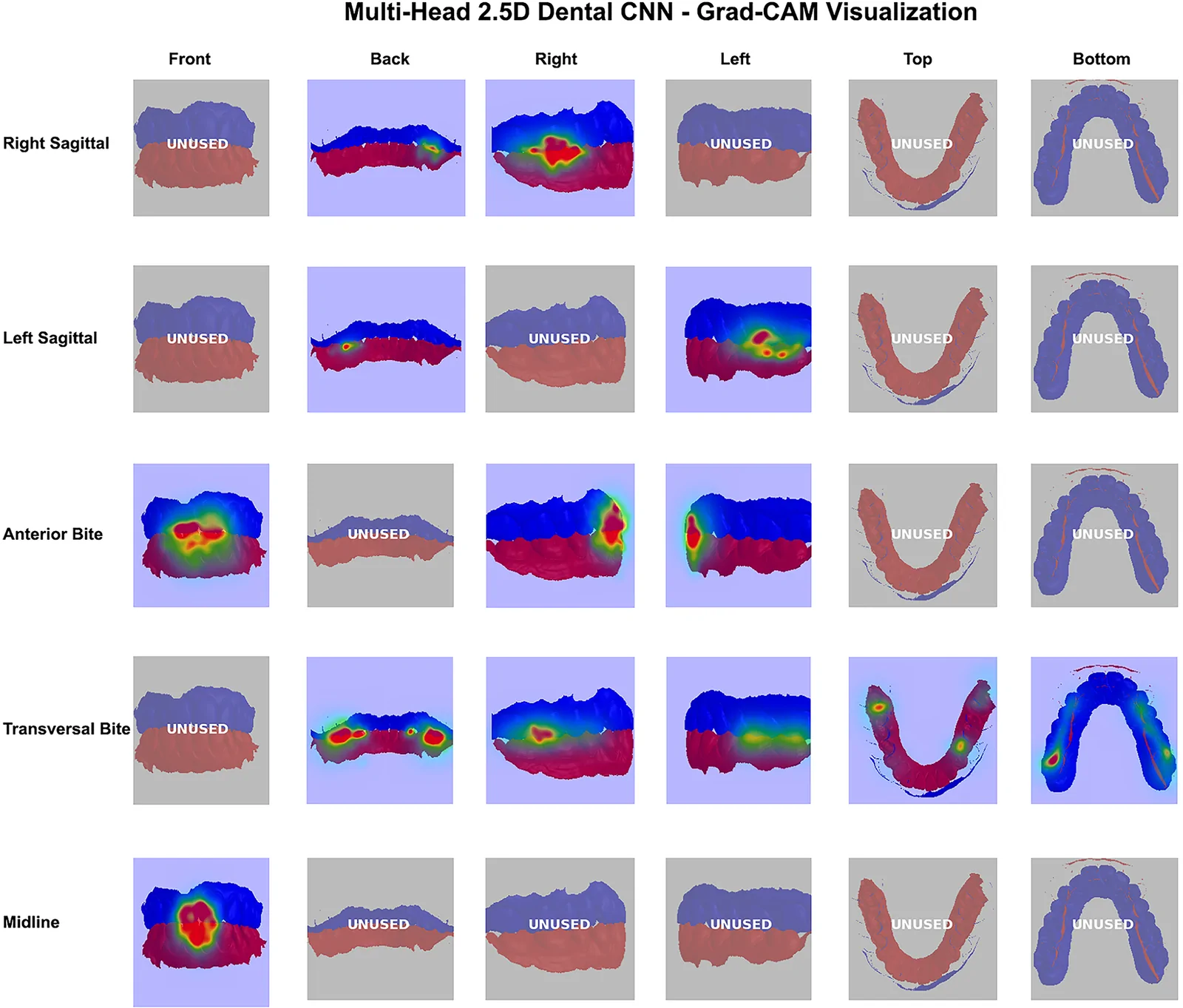
Grad-CAM visualizations illustrating the regions of interest used by the multi-task model across different diagnostic heads and input views. Warmer colors (red/yellow) indicate higher contribution to the model’s decision. For each task, only the views actively used by the corresponding head are highlighted, while unused views are indicated accordingly

To improve the interpretability of the proposed model, Gradient-weighted Class Activation Mapping (Grad-CAM) was applied to visualize the regions contributing to the classification decisions (Fig. 5). The results demonstrate that the model consistently focuses on clinically relevant anatomical regions for each task. For sagittal classification, attention is primarily concentrated around posterior occlusal relationships, particularly the molar cusp alignment regions. In anterior bite classification, the model emphasizes the vertical overlap between maxillary and mandibular incisors. For transversal occlusion, attention is distributed along the dental arch, especially in occlusal views, reflecting buccolingual relationships. In midline classification, the model predominantly focuses on the anterior region where dental midline alignment is assessed. These findings suggest that the model does not rely on arbitrary image features but instead captures clinically meaningful patterns consistent with orthodontic diagnostic principles.

### Sagittal molar relationships

For right sagittal molar relationship classification, the highest accuracy values were achieved by the VGG16 (72.0%) and ResNet50 (71.3%) models. Examination of ROC-AUC values revealed that ResNet50 exhibited strong discriminative ability (ROC-AUC ≈ 0.88). In left sagittal classification, the ResNet50 model demonstrated the most stable performance, achieving 69.4% accuracy along with balanced sensitivity and specificity values. In contrast, the performance of the VGG16 and MobileNetV2 architectures in left sagittal classification showed a marked decline.

### Anterior bite classification

In anterior bite classification (normal bite, deep bite, and open bite), the EfficientNetV2B3 architecture achieved the best performance, with 80% accuracy, an F1-score of 0.81, and a high ROC-AUC value (≈ 0.89). This finding indicates that the EfficientNetV2B3 architecture is more effective in distinguishing vertical occlusal relationships. Accuracy values for the other architectures ranged between 44% and 65%, with particularly low F1-scores observed for the ConvNeXtTiny and MobileNetV2 models.

### Transversal occlusal relationships

In transversal occlusion classification, the performance of all models was lower compared with the other tasks. The highest accuracy values were obtained with the VGG16 (68.0%) and ResNet50 (63.1%) models. The relatively low ROC-AUC values indicate that distinguishing transversal relationships using multi-view 2D representations is more challenging. This may be related to the greater dependence of transversal anomalies on three-dimensional depth information.

### Midline classification

For midline evaluation, models generally demonstrated higher performance. The MobileNetV2 (80.0%) and ResNet50 (78.8%) models achieved high accuracy values. The ResNet50 model yielded the most balanced results for detecting midline deviation, with high sensitivity (83.7%) and an F1-score of 0.86. This finding suggests that midline assessment provides more prominent morphological cues in multi-view images.

## Discussion

In this study, we aimed to simultaneously and multi-task classify the sagittal, vertical, and transverse components of malocclusion, as well as midline status, using multi-view 2D representations derived from three-dimensional IOS data. Although artificial intelligence applications in orthodontic diagnosis have increased rapidly in recent years, the majority of existing studies have focused on a single imaging modality, a single diagnostic task, and predominantly on 2D data [8, 16–18]. In this context, the present study is both directly comparable to and complementary with the existing literature in terms of its methodological approach and scope.

Previous studies have reported high accuracy rates in malocclusion classification using intraoral photographs [8, 16]. However, such 2D approaches are limited in capturing occlusal relationships and transverse discrepancies. In contrast, IOS-based data provide more comprehensive three-dimensional morphological information. Similarly, cephalometric-based AI studies have shown strong performance; however, they are limited by projection errors and lack detailed information on dental arch form and transverse relationships [17–20]. IOS-based approaches overcome some of these limitations by directly utilizing dental surface morphology.

With respect to STL-based studies, Juneja et al. performed occlusion classification using 2D projections derived from intraoral scans and achieved an accuracy of 84.39% with their best model [9]. However, that study focused solely on Angle classification, excluding vertical and transverse dimensions as well as midline assessment. Similarly, a substantial proportion of STL-based studies in the literature have concentrated on single-task classification or on segmentation and tooth alignment problems [17, 21]. While the multi-task structure of the present study provides a scenario closer to real clinical diagnostic processes, the additional performance burden imposed by this approach is also clearly reflected in the results.

The relatively lower accuracy values obtained for sagittal molar relationship classification compared with some photograph- or cephalogram-based studies are noteworthy. One possible explanation is that sagittal molar relationships can only be indirectly inferred from IOS data based on crown morphology and contact relationships. In clinical practice, sagittal assessment is often performed in conjunction with cephalometric skeletal references and soft tissue analysis [18, 19]. Therefore, it is expected that a model relying solely on dental surface data may show limited performance, particularly in borderline cases. Nevertheless, the relatively high ROC-AUC values indicate that the model was able to capture discriminative morphological signals between classes.

The superior performance of the EfficientNetV2B3 architecture in anterior bite classification is consistent with the presence of more distinct geometric cues for vertical occlusal relationships in IOS data. Considering that overbite and open bite assessments in the literature are often based on manual measurements [8, 20], these findings support the clinical potential of IOS-based automated systems. However, some studies have reported higher error rates for AI models compared with clinicians in overbite measurements [8], suggesting that vertical relationships should be evaluated not only using static occlusal images but also in conjunction with functional and skeletal factors.

The relatively low performance observed across all models for transverse occlusion classification is consistent with similar findings reported in the literature. The diagnosis of transverse anomalies depends on multiple parameters, including arch width, alveolar bone relationships, and functional shifts [9, 17]. Although multi-view 2D representations partially preserve this information, approaches that directly utilize 3D point clouds or volumetric data may offer advantages for this task. Nevertheless, the high computational cost and clinical integration challenges associated with 3D CNN or point cloud-based models suggest that the multi-view approach provides a practical balance between performance and feasibility.

The high performance achieved in midline classification can be explained by the presence of clear and localized morphological cues in frontal and occlusal views. Similarly, studies based on intraoral photographs have reported higher accuracy for midline assessment compared with other tasks [8, 16]. The use of IOS data further reduces the influence of factors such as head–camera positioning or photographic angle, contributing to more robust evaluations. From a clinical perspective, diagnostic agreement in orthodontics is known to show moderate to substantial variability among clinicians, particularly for complex cases. In this context, the performance achieved in this study—especially for anterior bite and midline classification—can be considered clinically meaningful. However, the model is intended as a decision-support tool rather than a replacement for clinical judgment.

Although explicit millimeter-based measurements were not directly used in the analysis, the classification labels were assigned based on standard clinical orthodontic evaluation, which inherently reflects underlying quantitative relationships such as overbite and midline deviation. Therefore, the model’s probabilistic outputs can be interpreted as indirectly capturing these clinically meaningful quantitative features. Future studies incorporating explicit numerical measurements may further strengthen this relationship and provide additional validation.

Several limitations of this study should be acknowledged. First, the dataset size is relatively limited for deep learning models, and class imbalance exists in certain categories. This may have negatively affected performance, particularly for transverse occlusion and some sagittal classes. Second, the multi-view 2D representation does not fully capture all three-dimensional information; therefore, comparative studies with direct 3D point cloud or mesh-based approaches are warranted. Third, the model relies solely on static occlusion data, excluding functional occlusion, dynamic movements, and soft tissue effects. In addition, the dataset included scans obtained from different intraoral scanner systems, which may introduce variability in image quality and geometry. Although this heterogeneity may enhance model generalizability, it could also affect performance consistency. Although the proposed architecture assigns specific view combinations to each diagnostic task based on clinical relevance, no explicit ablation study was conducted to quantitatively evaluate the contribution of individual views or alternative view combinations. The selection of views was guided by orthodontic diagnostic principles, where certain anatomical perspectives are known to be more informative for specific tasks. Future studies could systematically investigate the impact of different view combinations through ablation analyses to further optimize model performance and improve interpretability, particularly for tasks such as transverse occlusion classification, where performance was comparatively lower.

## Conclusion

This study demonstrates that the sagittal, vertical, and transverse components of malocclusion, as well as midline status, can be simultaneously and automatically classified using multi-view images derived from three-dimensional intraoral scan data. The proposed multi-task deep learning model achieved high performance particularly in anterior bite and midline evaluation, while producing clinically meaningful yet improvable results for sagittal and transverse relationships.

These findings indicate that IOS-based artificial intelligence applications have the potential to enhance objectivity in orthodontic diagnosis, accelerate clinical workflows, and serve as supportive decision-making tools in treatment planning. Future studies incorporating direct 3D representations or hybrid models combining multi-view and point cloud approaches may further improve performance, especially in the assessment of transverse anomalies. Additionally, studies conducted with larger, multicenter, and longitudinal datasets will strengthen the clinical applicability and reliability of such systems.

## Supplementary Information

Below is the link to the electronic supplementary material.

Supplementary Material. Training and validation loss curves across epochs for each output head of the multi-task model, including right sagittal, left sagittal, anterior bite, transverse bite, and midline classification tasks

## Data Availability

The data and materials used in the study can be obtained by contacting the corresponding author.
